# Long-range interactions, wobbles, and phase defects in chains of model cilia

**DOI:** 10.1103/PhysRevFluids.1.081201

**Published:** 2016-12-13

**Authors:** Douglas R. Brumley, Nicolas Bruot, Jurij Kotar, Raymond E. Goldstein, Pietro Cicuta, Marco Polin

**Affiliations:** 1 Ralph M. Parsons Laboratory, Department of Civil and Environmental Engineering, Massachusetts Institute of Technology, Cambridge, Massachusetts 02139, USA; 2 Department of Civil, Environmental and Geomatic Engineering, ETH Zürich, 8093 Zürich, Switzerland; 3 Institute of Industrial Science, University of Tokyo, 4-6-1 Komaba, Meguro-ku, Tokyo 153-8505, Japan; 4 Cavendish Laboratory, University of Cambridge, Cambridge CB3 0HE, United Kingdom; 5 Department of Applied Mathematics and Theoretical Physics, University of Cambridge, Centre for Mathematical Sciences, Wilberforce Road, Cambridge CB3 0WA, United Kingdom; 6 Physics Department, University of Warwick, Gibbet Hill Road, Coventry CV4 7AL, United Kingdom

## Abstract

Eukaryotic cilia and flagella are chemo-mechanical oscillators capable of generating long-range coordinated motions known as metachronal waves. Pair synchronization is a fundamental requirement for these collective dynamics, but it is generally not sufficient for collective phase-locking, chiefly due to the effect of long-range interactions. Here we explore experimentally and numerically a minimal model for a ciliated surface: hydrodynamically coupled oscillators rotating above a no-slip plane. Increasing their distance from the wall profoundly affects the global dynamics, due to variations in hydrodynamic interaction range. The array undergoes a transition from a traveling wave to either a steady chevron pattern or one punctuated by periodic phase defects. Within the transition between these regimes the system displays behavior reminiscent of chimera states.

The ability of ensembles of oscillators to achieve collective motions is fundamental in biological processes ranging from the initiation of heartbeats to the motility of microorganisms. The emergent properties of coupled oscillators can vary dramatically depending on the intrinsic properties of the oscillators and the nature of the coupling between them [1]. Flashing fireflies equally and instantaneously coupled to one another [2] can support very different behaviors to chemical micro-oscillators, which are coupled only locally, and subject to time delays [3].

Eukaryotic cilia and flagella are chemo-mechanical oscillators that generate a variety of collective motions, which can be quantified with high-speed imaging in microfluidic environments [4–6]. The molecular biology of these internally driven filaments is virtually identical in green algae [5], protists [7], and humans [8], and the flows they generate fulfill crucial roles in development, motility, sensing, and transport. When close together, the mutual interaction between their oscillatory flow fields can cause them to beat in synchrony [9], and larger ensembles of flagella demonstrate striking collective motions in the form of metachronal waves (MWs) [10–13], akin to the “Mexican wave” propagating around a packed stadium. Many surrogate models for flagellar dynamics have been proposed [13–24], typically with a set geometry that fixes the range and coupling between oscillators.

Here we relax this condition and study a linear array of colloidal oscillators [25] driven in circular trajectories at a controllable height above a no-slip wall. Originally introduced as a mathematically convenient minimal model for synchronization at low Reynolds numbers [15], colloidal rotors have been experimentally shown to reproduce the time-dependent flow field associated with a beating flagellum down to distances comparable to its size (~10 *μ*m) [9,26]. When generalized to include waveform flexibility [14,27,28] they are also capable of capturing interflagellar synchronization in bulk [9]. The system of colloidal rotors studied here can be modified continuously from being primarily coupled through nearest neighbors to a regime involving significant long-range interactions. As a function of rotor properties, a traveling wave found at small heights becomes either a chevron pattern or is punctuated by phase defects at large ones. The transition is not a gradual morphing between the two profiles, but rather a process involving generation and propagation of defects along the strip, where phase-locked and non-phase-locked subgroups of oscillators can coexist. A behavior arising from long-range interactions whose amplitude is modulated by the distance from the wall [18], these dynamics are reminiscent of chimera states, in which oscillators split into phase-locked and desynchronized clusters [29,30].

In our experiments, silica colloids of radius *a* = 1.74 *μ*m (BangsLab, USA) suspended in a water-glycerol solution of viscosity *μ* = 6 mPas within a 150-*μ*m-thick sample, are captured and driven by feedback-controlled time-shared (20 kHz) optical tweezers (OTs) based on acousto-optical deflection of a 1064-nm-wavelength diode-pumped solid-state laser (CrystaLaser IRCL-2W-1064) as previously described [31,32]. The OTs describe a planar array of circular trajectories [Fig. 1(a)] of radius *R* = 1.59 *μ*m and center-to-center separation *ℓ* = 9.19 *μ*m, a distance *h* above the sample bottom, with 4.2(4) ⩽ *h* ⩽ 51.7(4) *μ*m. This configuration, which reflects the capabilities and limitations of our OT setup, is similar to arrays of nodal cilia, but differs from another common situation where the ciliary beating plane is perpendicular to an organism’s surface.

The oscillators are imaged using a Nikon inverted Eclipse Ti-E with a 60× Nikon Plan Apo VC water immersion objective (NA = 1.20), and recorded for up to 1200 s using an AVT Marlin F131B CMOS camera set at 229 fps. The rotor positions are measured using an algorithm that correlates the image intensity *I* (*x, y*) with a rotationally symmetric kernel image *K*(*x, y*) constructed from a real colloid. By fitting the two-dimensional (2D) cross-correlation function *C*(*x*_0_,*y*_0_) = ∑_(*x,y*)_ *I* (*x,y*) × *K*(*x* − *x*_0_, *y* − *y*_0_) with a 2D parabola and maximizing this function, the rotor positions (*x*_0_, *y*_0_) are extracted with subpixel resolution and used to track their phases {*ϕ_i_*(*t*)} over time [Fig. 1(a)].

The rapid feedback loop between colloid and trap positions facilitates the arbitrary placement of the OTs with respect to the colloidal particles. The trap positions are maintained at a constant radius *R* and fixed angular distance ahead of the colloids. Consequently, a colloidal particle on the *i*th trajectory (*i* ∈ {0, …, *N* − 1}) experiences a radial harmonic potential with spring constant *λ* = 2.06 ± 0.06 pN/*μ*m resisting excursions from the prescribed radius, and a constant tangential force of magnitude *F_i_* = *F*_dr_*D*^*i*−0.5^ leading to rotation. The period of rotation is therefore not fixed, and it is this degree of freedom that permits synchronization of interacting particles. The choice of *λ* reflects estimates of the bending rigidity of flagella, *κ* ~ 4 × 10^−22^ N m^2^, and their length, *L*. From *λ* = *κ/L*^3^ [14], and for typical values of *L*, values of λ~𝒪(1–10)pN/μm should represent typical flagella. Unavoidable delays in the OT’s feedback response introduce a mismatch between the parameters used in experiments and simulations, which is corrected by increasing the simulation value of *λ* by a constant factor *γ* relative to the experimental one. The previously reported value of *γ* = 2.21 [32] is adopted throughout this paper, which results in quantitative agreement of simulations with the present experiments [see Fig. 1(c)].

Isolated oscillators rotate with a height-dependent angular velocity *ω_i_* = *F_i_/Rζ*_0_*ζ_w_*, where *ζ*_0_ = 6*π μa* is the sphere’s bulk drag coefficient, and $\zeta_{w}\left(h\right)=1+\frac{9}{16}\frac{a}{h}+𝒪\left(a^{3}/h^{3}\right)$ accounts for the presence of the wall [33]. Experimental results are compared with deterministic hydrodynamic simulations in which colloids are treated as point-like particles above a no-slip boundary, and therefore coupled through the so-called “Blake tensor” [11,34]. Before each experiment we calibrate *F*_dr_ ≃ 2.23 pN (see Supplemental Material [35]; typical variation ±2%). *D* ≠ 1 is used to break left-right symmetry along the chain and induce a stable traveling wave for small *h* [11]. For the detuning adopted here, *D* = 1.01, the period of individual oscillators varies between *τ* ~ 0.5 s and ~1 s across the explored range of *h*.

Consider first two rotors separated by a distance *ℓ*. For rotors with instantaneous positions {***x**_i_*} and velocities {***v**_i_*}, the hydrodynamic drag on the *i*th rotor is given by $-\zeta \left({\text{x}}_{i}\right)\cdot \left[{\text{v}}_{i}-\sum_{j\neq i}\text{G}\left({\text{x}}_{j},{\text{x}}_{i}\right)\cdot {\text{F}}_{j}^{\text{ext}}\right],$ where ${\text{F}}_{j}^{\text{ext}}$ is the net external force acting on the *j*th sphere and **G**(***x**_i_*,***x**_j_*) is the Green’s function in the presence of the no-slip wall. For identical rotors (detuning *D* = 1), hydrodynamic coupling eventually leads to synchrony provided *λ* < ∞, by perturbing the angular velocities of the two rotors so that the leading and lagging rotors become slower and faster respectively [13,14]. The timescale for synchronization is proportional to the spring constant *λ* [see Eq. (1)] and also depends on the strength of hydrodynamic interactions between rotors, which is a function of height *h* and spacing *ℓ*. The dynamics become richer if a discrepancy between the rotor’s intrinsic frequencies is introduced (*D* ≠ 1), for then the coupling must be sufficiently strong to overcome the natural tendency for the rotor’s phase difference *χ* = *ϕ*_1_ − *ϕ*_0_ to drift.

Bifurcation plots in Fig. 1(b) show, for different *h*, the average phase drift between two oscillators as a function of *D*. The behavior is typical of a saddle-node bifurcation: the oscillators phase-lock until *D* reaches a critical value D*(*h*) and then drift with a monotonically increasing speed. D*(*h*) increases with *h*, reflecting the strengthening of inter-rotor hydrodynamic coupling with increasing distance from the wall. The phase-locking behavior is summarized in Fig. 1(c), where the experimental synchronization boundary is based on a threshold of five slips in the whole experiment $({\dot{\chi }}_{\text{av}}=0.131\;\text{rad}/\text{s}).$ The results of individual experiments are classified based on this threshold, and are represented as either red (drift) or blue (synchronized) points in Fig. 1(c). As *h* is increased, the rotor pair moves deeper into the synchronized region: the coupling between the two strengthens due to lower hydrodynamic screening from the wall, leading to an enhanced stability of the synchronized state. This is reproduced by simulations [Fig. 1(c)] up to small shift in *D*, which could come from the finite value of *a/h* and experimental noise. In the limit *a,R* ≪ *ℓ*, the evolution of the phase difference *χ* = *ϕ*_1_ − *ϕ*_0_ can be derived by a generalization of previous arguments [14,35]. As phase-locking is slow compared to the rotation period, we average over this fast timescale and find

(1) $$\dot{\chi }=\frac{F_{1}-F_{0}}{R_{0}\zeta_{0}\zeta_{w}}-\frac{3a}{4\ell }\frac{F_{0}F_{1}}{\lambda \zeta_{0}R_{0}^{2}}\left[2A\left(\beta \right)+B\left(\beta \right)\right]\;\text{sin}\;\chi ,$$

where $A\left(\beta \right)=1-X-\frac{\beta^{2}}{2}X^{3},\;B\left(\beta \right)=1-X^{3}+\frac{3\beta^{2}}{2}X^{5},\;X=1/\sqrt{1+\beta^{2}},\;\text{and}\;\beta =2h/\ell .$ From Eq. (1), the average phase drift ${\dot{\chi }}_{\text{av}}$ for non-phase-locked states reads

(2) $${\dot{\chi }}_{\text{av}}=\sqrt{{\left(\frac{F_{1}-F_{0}}{R_{0}\zeta_{0}\zeta_{w}}\right)}^{2}-{\left(\frac{3a}{4\ell }\frac{F_{0}F_{1}}{\lambda \zeta_{0}R_{0}^{2}}\left[2A\left(\beta \right)+B\left(\beta \right)\right]\right)}^{2}.}$$

Given the functional form of the frequency detuning, *F_i_* = *F*_dr_*D*^*i*−0.5^, Eq. (2) can be solved explicitly to yield the critical detuning D*(*h*) [solid line in Fig. 1(c)]. The theoretical and numerical solutions for the boundary in Fig. 1(c) slightly under- and overestimate the data, respectively, owing to neglect of temporal variations in the interparticle spacing and the finite size of the beads, respectively. Both also neglect thermal fluctuations.

We now turn to the dynamics of a linear array of six rotors, with the *i*th rotor centered at ***x*** = (*il*,0,*h*). This is the longest controllable chain with our active-feedback-based OTs. Linear arrays of colloidal oscillators have been shown to capture the dynamics of two-dimensional arrays [13], so this simplified geometry will be the focus here. The dynamics are studied experimentally as a function of *h*, but numerical simulations allow wider exploration of parameters, including changes in the radial stiffness *λ*, which governs the coupling strength [9,11,13,14,32] as in Eq. (1). In both experiments and simulations we introduce a mild frequency bias *D* = 1.01, typical also of *Volvox* colonies [13], which breaks the translational symmetry and induces a MW for *h* ≲ 10 *μ*m. At all heights studied, this value of *D* is deep within the synchronized region of parameter space for two rotors.

Figure 2(a) shows that at *h* = 6.7(4) *μ*m the rotors phase-lock in a stable MW whose direction is set by the frequency bias. With increasing *h*, defects (phase slips) emerge, giving rise to a net drift in the cumulative phase difference between rotors at opposite ends of the chain. Phase defects always propagate in the direction of the fastest oscillator. At these intermediate heights, the phase profile also displays “wobbles,” perturbations to the MW that are not accompanied by a phase defect. Numerical results shown in Fig. 2(b) capture the traveling wave at *h* = 5 *μ*m, the presence of defects and their propagation direction, and wobbles at larger heights. At the largest height, *h* = 50 *μ*m, defects no longer propagate through the chain, and rotors 3–5 remain phase-locked.

The phase dynamics of wobbles and defects are shown in Fig. 3(a) for *h* = 11.7(4) *μ*m. The first 25 seconds of the time series show fluctuations in *ϕ_i_* − *ϕ*_0_ (wobbles), even while the system is frequency locked. Fluctuations start at the first oscillator pair and travel unidirectionally along the chain [Fig. 3(b)] with a preserved, soliton-like signature [36]. Occasionally, they terminate within the chain with a slip [Fig. 3(a)]; these are the phase defects observed in kymographs. Both wobbles and defects are characterized by initial excursions of amplitude *W* and recurrence time *τ* [Fig. 3(c)], which depend on *h* [Figs. 3(d) and 3(e)]. The typical time *τ* ~ 10 〈*T*〉 (where 〈*T*〉 ≃ 1 s is the average period) depends less strongly on *h* than does *W*, which shows a pronounced growth [Fig. 3(d)], mirroring the increased probability that a wobble will terminate in a slip within the chain, causing a defect [Fig. 3(f)]. Although their position can vary, defects tend to cluster, in this case at the middle of the chain (position *i* = 2), as seen also in simulations of longer chains [35].

The hydrodynamic coupling between two rotors increases monotonically with *h*; for an isolated pair, this manifests in more robust synchronization at larger heights. For a chain of rotors, increasing *h* has the reverse effect, disrupting the stable MW with wobbles and punctuating it with periodic phase defects (Fig. 2). The hydrodynamic coupling between every pair of rotors in the chain grows as *h* is increased. For just two rotors, Eq. (1) shows that equivalent changes to the hydrodynamic coupling can be achieved through modification of the mean interparticle separation *l*. For the chain of six rotors, in which longer range hydrodynamic interactions also occur, changes to *h* and *l* are no longer equivalent.

The peculiar dynamics observed arise from a change in the relative contributions of interactions with different neighbors. The no-slip wall has the effect of screening the hydrodynamic interactions in a way that qualitatively changes as a function of *β* = 2*h/ℓ*. This is an important determinant of MW stability, as observed also in simulations of colloidal “rowers” [18]. Figure 2(c) shows the magnitude of the coupling of a given oscillator with its *n*th nearest neighbor, estimated with Eq. (1), normalized by the total interaction strength with the first five neighbors. Although all pairwise couplings grow monotonically with *h*, the relative magnitude of the nearest neighbor interactions actually diminishes. Conversely, the relative importance of all others increases with *h*. Hydrodynamic disturbances parallel to the wall decay as *u* ~ *r*^−*j*^ where *j* = 1 and 3 represent the far (*β* ≫ 1) and near (*β* ≪ 1) asymptotic limits [18]. For the end rotor the magnitude of the coupling with the *n*th nearest neighbor, normalized by the total coupling strength, is $S\left(n\right)=n^{-j}/\sum_{i=1}^{5}i^{-j}.$ For *β* ≪ 1 the interactions are dominated by nearest neighbor, with *S*(1) = 0.84, while for *β* ≫ 1, *S*(1) = 0.44 [see the black curve in Fig. 2(c)]. We test the hypothesis that the breakdown of the traveling wave is due to long-range hydrodynamic interactions through simulations in which interactions are truncated at nearest neighbors, and find the abundance of defects is significantly reduced. Importantly, the dynamics are nearly insensitive to *h*, with a maximum relative variation in end-to-end drift speed of just 3% between *h* = 5 *μ*m and 1000 *μ*m (Fig. 4) [35].

Additional numerical simulations permit the wider exploration of parameter space. The range of *λ* values studied here corresponds to the estimated values *λ* = *κ/L*^3^ based upon ciliary lengths of *L* ~ 4−10 *μ*m [14]. Figure 4(a) shows the average end-to-end phase drift per beat as a function of *λ* and *h*, and enables analysis of many numerical simulations without looking at the individual kymographs. The area of solid blue corresponds to specific parameter combinations for which complete phase-locking occurs. However, from the drift alone, one cannot distinguish between a linear traveling wave ③ and a chevron phase profile ①. For this we compute the complex order parameter $Z=Ae^{i\text{Ψ}}=\frac{1}{N-1}\sum_{n=0}^{N-1}e^{i\chi_{n}}$ where *χ_n_* = *ϕ*_*n*+1_ − *ϕ_n_* [18,37]. Note the use of the pairwise phase differences, not the individual rotor phases. Looking at the mean value of $\left|\bar{\Psi }\right|$ [see Fig. 4(c)], the region of parameter space corresponding to complete phase-locking can be decomposed into chevron $\left(|\bar{\Psi }|\approx 0\right)$ and MW $\left(|\bar{\Psi }|>0\right)$ regions. Using the average values *Ā* and $\bar{\Psi }$ for *t* > 200 s [Figs. 4(b) and 4(c)], we see that as *h* is increased at the experimental value of *λ* (white dotted line), the stable traveling wave at small *h* shifts to a profile with defects and wobbles, initially along the whole chain, and then localized to one-half of the chain with the remaining three oscillators constantly phase-locked.

At values of *λ* smaller than the experimental one, however, we observe qualitatively different dynamics. For *λ* ≲ 2.5 pN/*μ*m, the pattern morphs continuously between different types of complete synchronization as *h* is increased, going from a MW ③ to a chevron-like pattern ①. These transitions happen without the emergence of defects [11]. For 2.5 < *λ* < 3 pN/*μ*m the system shows reentrant behavior with defects only at intermediate heights, separating a MW region from a chevron-like region. The order parameter angle $\left|\bar{\Psi }\right|$ [Fig. 4(c)] identifies clearly the stable MW (yellow/orange) and chevron (dark blue) regions of parameter space. For a fixed *h* ≳ 50 *μ*m, increasing *λ* results in a monotonic decrease in *Ā* owing to the reduced rotor compliance. Conversely, the end-to-end phase drift exhibits a strong peak around *λ* = 4.5 pN/*μ*m, where the rotors slip approximately one beat in every five, despite an intrinsic frequency difference of just 5%. These nontrivial dynamics emerge due to the combination of phase slips induced by long-range interactions, and rapid healing of phase defects through orbit compliance. The complete absence of these features from the simulations with nearest neighbor coupling alone [Fig. 4(d)] highlights the role played by competition between interactions at different ranges. Changing *h* is then a simple and accessible way to modulate their relative strength (see Fig. 2).

Large arrays of cilia are synonymous with no-slip boundaries, and in many cases, the spacing between these organelles is comparable to their length [13], so that effectively *h*/*ℓ* ~ 1 [see Fig. 4(a)]. Our results suggest that flagella of *Volvox* may then be balancing the need to extend out into the fluid enough to generate a vigorous thrust, with the screening of long-rage hydrodynamic interactions necessary to stabilize MWs on the colony surface. As a result, ensembles of flagella in *Volvox* [11] (but see also numerical simulations [22]) may operate in a regime naturally prone to the emergence of metachronal phase defects, which are indeed observed experimentally [13].

## Supplementary Material

Supporting information

## Figures and Tables

**Fig. 1:**
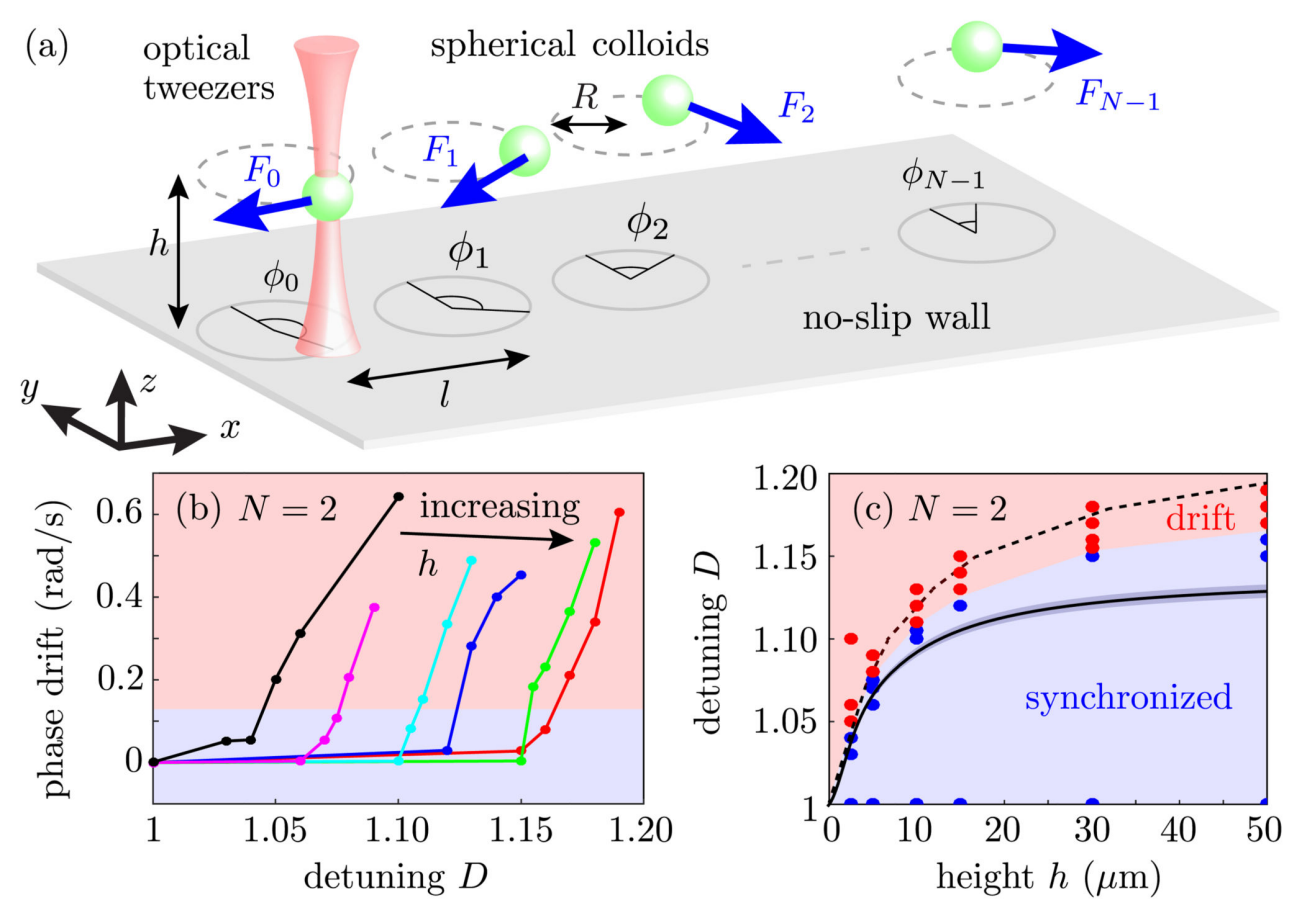
Experimental setup and results. (a) Microspheres of radius *a* = 1.74 *μ*m, situated at a distance *h* above a no-slip boundary are driven by time-sharing optical tweezers in circular trajectories of radius *R* = 1.59 *μ*m and center-to-center separation *ℓ* = 9.19 *μ*m. (b) Average phase drift $\dot{\chi }={\dot{\phi }}_{1}-{\dot{\phi }}_{0}$ for a rotor pair vs detuning *D* for *h* = 4.2 (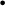), 6.7 (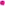), 11.7 (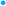), 16.7 (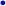), 31.7 (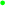), 51.7 (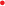) *μ*m. (c) Phase diagram showing experimental regions of synchrony (blue) and drift (red), the boundary from hydrodynamic simulations (dashed), and theory from Eq. (2) (solid).

**Fig. 2:**
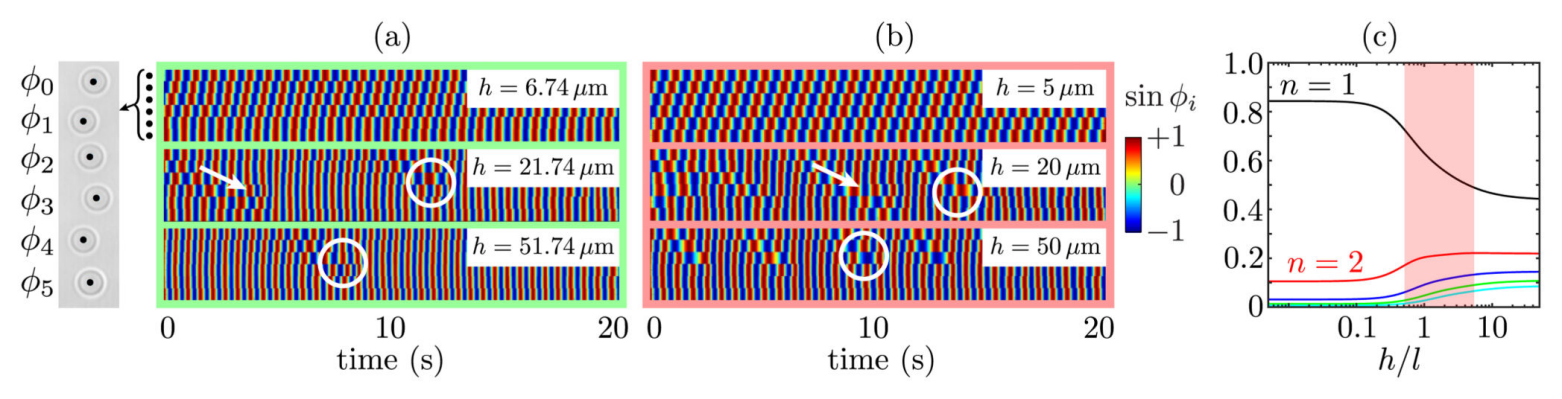
Results for the linear array of driven colloidal oscillators, shown schematically in grey (not to scale). (a) Kymographs showing sin *ϕ_i_* at three heights above the wall. With increasing *h*, the traveling wave becomes frustrated, with the introduction of wobbles (arrows) and phase defects (circles). (b) Numerical results from model. (c) Fraction of total coupling corresponding to interacting with different neighbors, as a function of *h*. The shaded red region represents the experimental parameter regime.

**Fig. 3:**
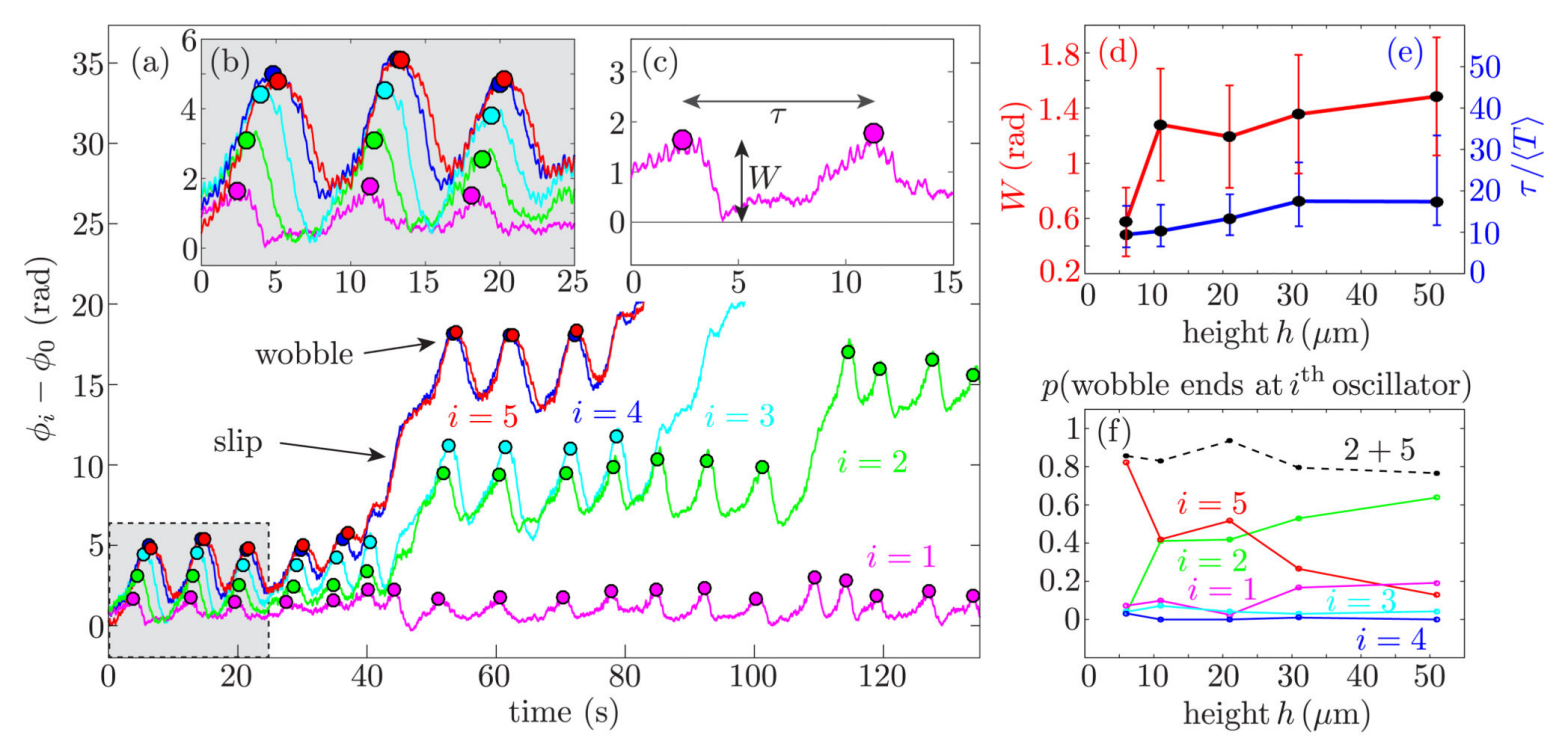
Experimental phase dynamics. (a),(b) Phase difference relative to the first rotor, *ϕ_i_* − *ϕ*_0_, at *h* = 11.7 *μ*m. (c) Wobbles are characterized by their magnitude *W* (radians) and timescale *τ/*〈*T*〉 (normalized by rotor period), shown as a function of *h* in panels (d) and (e). (f) Probability that a propagating wobble ends at rotor *i*, resulting in a slip.

**Fig. 4:**
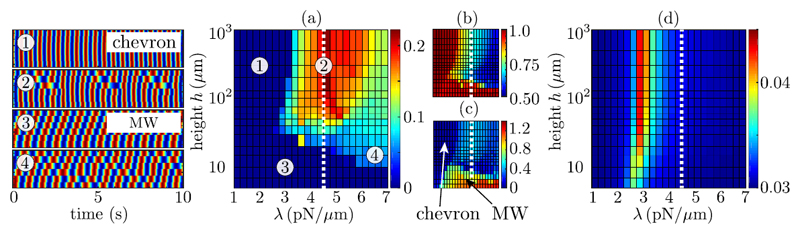
(a) Average phase drift per beat between end oscillators (measured in beats) as a function of height above the wall and radial spring stiffness. Shown also are four representative kymographs. (b) Time-averaged amplitude *Ā* and (c) angle $\left|\bar{\Psi }\right|$ of the complex order parameter *Z* = *Ae*^*i*Ψ^. The axes are the same as in (a). (d) Same as (a) but with hydrodynamic interactions truncated to nearest neighbor. Parameters used include *a* = 1.74 *μ*m, *ℓ* = 9.19 *μ*m, *R* = 1.59 *μ*m, and viscosity *μ* = 6 mPas. Simulations correspond to 0 ≤ *t* ≤ 2000 s. The dashed white line shows the value of *λ* corresponding to Fig. 2. Note the different color scales used throughout.

## References

[1] Dörfler F, Bullo F (2014). Synchronization in complex networks of phase oscillators: A
survey. Automatica.

[2] Mirollo RE, Strogatz SH (1990). Synchronization of pulse-coupled biological
oscillators. SIAM J Appl Math.

[3] Toiya M, González-Ochoa HO, Vanag VK, Fraden S, Epstein IR (2010). Synchronization of chemical micro-oscillators. J Phys Chem Lett.

[4] Son K, Brumley DR, Stocker R (2015). Live from under the lens: Exploring microbial motility with
dynamic imaging and microfluidics. Nat Rev Micro.

[5] Goldstein RE (2015). Green algae as model organisms for biological fluid
dynamics. Annu Rev Fluid Mech.

[6] Quaranta G, Aubin-Tam ME, Tam D (2015). Hydrodynamics Versus Intracellular Coupling in the
Synchronization of Eukaryotic Flagella. Phys Rev Lett.

[7] Sleigh MA (1962). The Biology of Cilia and Flagella.

[8] Button B, Cai L, Ehre C, Kesimer M, Hill DB, Sheehan JK, Boucher RC, Rubinstein M (2012). A periciliary brush promotes the lung health by separating the
mucus layer from airway epithelia. Science.

[9] Brumley DR, Wan KY, Polin M, Goldstein RE (2014). Flagellar synchronization through direct hydrodynamic
interactions. eLife.

[10] Knight-Jones EW (1954). Relations between metachronism and the direction of ciliary beat
in Metazoa. Quart J Microsc Sci.

[11] Brumley DR, Polin M, Pedley TJ, Goldstein RE (2012). Hydrodynamic Synchronization and Metachronal Waves on the Surface
of the Colonial Alga Volvox Carteri. Phys Rev Lett.

[12] Elgeti J, Winkler RG, Gompper G (2015). Physics of microswimmers–single particle motion and
collective behavior: A review. Rep Prog Phys.

[13] Brumley DR, Polin M, Pedley TJ, Goldstein RE (2015). Metachronal waves in the flagellar beating of
*Volvox* and their hydrodynamic origin. J R Soc Interface.

[14] Niedermayer T, Eckhardt B, Lenz P (2008). Synchronization, phase locking, and metachronal wave formation in
ciliary chains. Chaos.

[15] Vilfan A, Jülicher F (2006). Hydrodynamic Flow Patterns and Synchronization of Beating
Cilia. Phys Rev Lett.

[16] Vilfan M, Potočnik A, Kavčič B, Osterman N, Poberaj I, Vilfan A, Babič D (2010). Self-assembled artificial cilia. Proc Natl Acad Sci USA.

[17] Uchida N, Golestanian R (2011). Generic Conditions for Hydrodynamic
Synchronization. Phys Rev Lett.

[18] Wollin C, Stark H (2011). Metachronal waves in a chain of rowers with hydrodynamic
interactions. Eur Phys J E.

[19] Gueron S, Levit-Gurevich K (1999). Energetic considerations of ciliary beating and the advantage of
metachronal coordination. Proc Natl Acad Sci USA.

[20] Cosentino Lagomarsino M, Jona P, Bassetti B (2003). Metachronal waves for deterministic switching two-state
oscillators with hydrodynamic interaction. Phys Rev E.

[21] Osterman N, Vilfan A (2011). Finding the ciliary beating pattern with optimal
efficiency. Proc Natl Acad Sci USA.

[22] Elgeti J, Gompper G (2013). Emergence of metachronal waves in cilia arrays. Proc Natl Acad Sci USA.

[23] Bruot N, Cicuta P (2013). Emergence of polar order and cooperativity in hydrodynamically
coupled model cilia. J R Soc Interface.

[24] Kavre I, Vilfan A, Babič D (2015). Hydrodynamic synchronization of autonomously oscillating
optically trapped particles. Phys Rev E.

[25] Bruot N, Cicuta P (2016). Realizing the physics of motile cilia synchronization with driven
colloids. Annu Rev Condens Matter Phys.

[26] Pedley TJ, Brumley DR, Goldstein RE (2016). Squirmers with swirl: A model for *Volvox*
swimming. J Fluid Mech.

[27] Elfring GJ, Lauga E (2011). Synchronization of flexible sheets. J Fluid Mech.

[28] Goldstein RE, Lauga E, Pesci AI, Proctor MRE (2016). Elastohydrodynamic synchronization of adjacent beating
flagella. Phys Rev Fluids.

[29] Abrams DM, Strogatz SH (2004). Chimera States for Coupled Oscillators. Phys Rev Lett.

[30] Martens EA, Thutupalli S, Fourrière A, Hallatschek O (2013). Chimera states in mechanical oscillator networks. Proc Natl Acad Sci USA.

[31] Leoni M, Kotar J, Bassetti B, Cicuta P, Lagomarsino MC (2009). A basic swimmer at low Reynolds number. Soft Matter.

[32] Kotar J, Debono L, Bruot N, Box S, Phillips D, Simpson S, Hanna S, Cicuta P (2013). Optimal Hydrodynamic Synchronization of Colloidal
Rotors. Phys Rev Lett.

[33] Happel J, Brenner H (1991). Low Reynolds Number Hydrodynamics.

[34] Blake JR (1971). A note on the image system for a Stokeslet in a no-slip
boundary. Math Proc Cambridge Philos Soc.

[R35] [35]See Supplemental Material at http://link.aps.org/supplemental/10.1103/PhysRevFluids.1.081201 for force calibration of individual rotors, analytical derivation of coupling in pairs, and supplementary numerical simulations of longer chains.

[36] Ahnert K, Pikovsky A (2008). Traveling waves and compactons in phase oscillator
lattices. Chaos.

[37] Pikovsky A, Rosenblum M, Kurths J (2003). Synchronization: A Universal Concept in Nonlinear Sciences.

