# Supplementary material for: Long-range interactions, wobbles, and phase defects in chains of model cilia
Source: Phys Rev Fluids. Author manuscript; Available in PMC 2018 Aug 17. (PMC6097603; doi:10.1103/PhysRevFluids.1.081201)
Supplement: Supporting information [file NIHMS78990-supplement-Supporting_information.pdf]

Long-range interactions and phase defects in chains of fluid-coupled oscillators Supplementary Material

Douglas R. Brumley[1,2], Nicolas Bruot[3,4], Jurij Kotar[4], Raymond E. Goldstein[5], Pietro Cicuta[4] and Marco Polin[6*]

The driving force on individual rotors is independent of height above the wall

For a single bead of radius a in a viscous fluid, situated at a distance h from an infinite no-slip boundary, the external applied force $\boldsymbol{F}$ is related to its velocity $\boldsymbol{v}$ according to $\boldsymbol{F} = \boldsymbol{\zeta} \cdot \boldsymbol{v}$, where $\boldsymbol{\zeta}$ is the anisotropic drag matrix, given by [1]

$$\boldsymbol{\zeta} = \boldsymbol{\zeta}(h) = \zeta_0 \left[\mathbf{I} + \tfrac{9a}{16h} (\mathbf{I} + \boldsymbol{e}_z \boldsymbol{e}_z) + \mathcal{O}((a/z)^3) \right]. \quad \text{(S1)}$$

The coefficient $\zeta_0 = 6\pi\mu a$ is the drag on the sphere in an unbounded fluid of viscosity μ (equivalent to setting $h \to \infty$). We are interested only in trajectories which are parallel to the no-slip wall ($\boldsymbol{v} \cdot \boldsymbol{e}_z = 0$). For a constant applied driving force $F_{\rm dr}$, the sphere's speed $v = |\boldsymbol{v}|$ is given by

$$v \simeq \frac{F_{\rm dr}}{\zeta_0 (1 + \frac{9}{16} \frac{a}{h})}, \quad \text{(S2)}$$

implying a monotonic increase of the sphere's speed with h for a given $F_{\rm dr}$. Each set of experiments involves studying the colloidal oscillators at a number of different heights h. For each set, the centre of the trajectory, its radius and the driving and radial forces are calibrated, for each individually loaded rotor, at the height of $h = 22\,\mu$m. These are then checked for independence on h. Figure S1 shows, after a full calibration, the speed of an individually loaded colloidal oscillator at 6 different heights together with the prediction from Eq. (S2) using a constant driving force. The two agree well for $F_{\rm dr} = 2.23\,$pN.

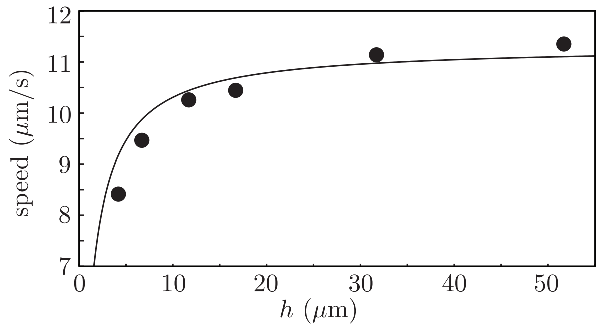

FIG. S1. Calibration of an individual colloidal oscillator, moving under the influence of a harmonic potential in an optical tweezer. Experimental results (dots) are shown alongside the prediction of Eq. (S2), with which the driving force of $F_{\rm dr} = 2.23$ pN can be extracted.

Single rotor force calibration Hydrodynamic interaction of two rotors at an arbitrary distance from a no-slip plane

The fluid disturbance produced by the motion of a sphere parallel to a no-slip wall depends on its height above the planar boundary. For two such spheres situated in the fluid, it is important to calculate the strength of the hydrodynamic interactions between them, and the subsequent effects on their dynamics. We consider two spheres of radius a driven around circular orbits of radius R_0 which are parallel to a no-slip wall. The orbit's centers are located at positions $(x, y, z) = (0, 0, h)$ and $(\ell, 0, h)$ respectively. The plane $z = 0$ represents the no-slip boundary, with the semi-infinite domain $z > 0$ filled with fluid of viscosity μ. $(\hat{e}_{\phi 1}, \hat{e}_{R1})$ and $(\hat{e}_{\phi 2}, \hat{e}_{R2})$ are the unit vectors of the local cylindrical frame of reference of each single rotor. This reference frame is centered at the center of the orbit. The displacement of each sphere from the center of its trajectory will be expressed in its cylindrical frame of reference as (R_1, ϕ_1) and (R_2, ϕ_2) respectively. Each rotor is subject to a constant tangential driving force $\boldsymbol{F}_i = F_i \hat{e}_{Ri}$, and also to a radial spring force with stiffness λ, which suppresses excursions from the equilibrium radius R_i. The spring stiffness is assumed to be large enough that the radial degree of freedom is slaved to the angular degree of freedom. That is, knowing (ϕ_1, ϕ_2), we know the instantaneous value of (R_1, R_2). It will be our goal to derive the equations of motion of the spheres, without making any assumptions about the relative magnitudes of ℓ and h.

We assume that sphere radius and trajectory radius are both small compared to other length scales ($a, R_0 \ll h, \ell$). Correspondingly, the two orbits are sufficiently far from each other that we can neglect the variation in the relative separation between the spheres as they move. The separation vector will always be taken to be $\ell \hat{e}_x$. We will write the relation between the sphere's angular velocity ω_i and the tangential driving force F_i, as $F_i = \zeta_0 \zeta_w R_0 \omega_i$, where $\zeta_0 = 6\pi\mu a$ is the bulk drag coefficient, and ζ_w is the correction due to the presence of the wall. For sufficiently small a/h this correction can be written as $\zeta_w = 1 + (9a/16h) + \mathcal{O}((a/h)^3)$. The first thing required is the generic expression of the 'Blakelet', i.e. the Stokeslet

on top of a bounding wall. This is given by [2]:

$$v_{21,i} = \frac{F_j}{8\pi\mu}\Bigg[\left(\frac{\delta_{ij}}{r} + \frac{r_i r_j}{r^3}\right) - \left(\frac{\delta_{ij}}{R} + \frac{R_i R_j}{R^3}\right) + 2h(\delta_{j\alpha}\delta_{\alpha k} - \delta_{j3}\delta_{3k})\frac{\partial}{\partial R_k}\left\{\frac{hR_i}{R^3} - \left(\frac{\delta_{i3}}{R} + \frac{R_i R_3}{R^3}\right)\right\}\Bigg] \tag{S3}$$

Here $\mathbf{r} = (\ell, 0, 0)$, $r = |\mathbf{r}|$; $\mathbf{R} = (\ell, 0, 2h)$, $R = |\mathbf{R}|$; $\alpha \in \{1, 2\}$. In our case, the point force will be in the (x, y) plane. Furthermore, we are only interested in the component of the velocity in the same plane, since the trajectories are constrained to lie at $z = h$. The background flow is due to the motion of the other sphere, which is:

$$\boldsymbol{v}_{21,||} = \frac{1}{8\pi\mu}\Bigg[\left(\frac{\mathbf{F}}{r} + \frac{\mathbf{r}(\mathbf{F}\cdot\mathbf{r})}{r^3}\right) - \left(\frac{\mathbf{F}}{R} + \frac{\mathbf{R}(\mathbf{F}\cdot\mathbf{R})}{R^3}\right)_{||} - 2h^2\left(\frac{\mathbf{F}}{R^3} - 3\frac{\mathbf{R}(\mathbf{F}\cdot\mathbf{R})}{R^5}\right)_{||}\Bigg]. \tag{S4}$$

In the limit as $h \to 0$, this reduces to Eq. (16) in [3]. Being interested only in the $||$ component, and because $\mathbf{F} = \mathbf{F}_{||}$, we can substitute

$$\mathbf{R}(\mathbf{F}\cdot\mathbf{R}) = (\mathbf{r} + 2h\hat{e}_3)(\mathbf{F}\cdot\mathbf{r}) \to \mathbf{r}(\mathbf{F}\cdot\mathbf{r}). \tag{S5}$$

Calling, as in [3], $\mathbf{s} = \mathbf{F}/8\pi\mu$ we get

$$\boldsymbol{v}_{21,||} = \frac{A(\beta)\mathbf{s} + B(\beta)\hat{r}(\mathbf{s}\cdot\hat{r})}{r} \tag{S6}$$

where $\hat{r} = \mathbf{r}/r = \hat{e}_x$, called $\mathbf{n}_{21}$ in [3]; $\beta = 2h/\ell$, and

$$A(\beta) = 1 - \left(\frac{1}{1+\beta^2}\right)^{\frac{1}{2}} - \frac{\beta^2}{2}\left(\frac{1}{1+\beta^2}\right)^{\frac{3}{2}} \tag{S7}$$

$$B(\beta) = 1 - \left(\frac{1}{1+\beta^2}\right)^{\frac{3}{2}} + \frac{3\beta^2}{2}\left(\frac{1}{1+\beta^2}\right)^{\frac{5}{2}} \tag{S8}$$

Notice that, due to the nearby wall, the strength of the Stokeslet $\mathbf{s}$ is written in terms of the sphere's velocity as:

$$\mathbf{s} = \frac{3}{4}a\zeta_0\zeta_w R_i\dot{\phi}_i\hat{e}_{\phi_i}. \tag{S9}$$

The derivation of the equations of motion follows the same procedure outlined in Appendix A of [3]. The only things we need to calculate are:

$$\hat{e}_{\phi 1}\cdot\boldsymbol{v}_{12} = \frac{3a}{8\ell}\zeta_w R_2\dot{\phi}_2[(2A(\beta) + B(\beta))\cos(\phi_1 - \phi_2) + B(\beta)\cos(\phi_1 + \phi_2)], \tag{S10}$$

$$\hat{e}_{R1}\cdot\boldsymbol{v}_{12} = \frac{3a}{8\ell}\zeta_w R_2\dot{\phi}_2[(2A(\beta) + B(\beta))\sin(\phi_1 - \phi_2) + B(\beta)\sin(\phi_1 + \phi_2)]. \tag{S11}$$

The rest of the calculation can be carried out in exactly the same way as in [3] and the final result is

$$\dot{\phi}_1 = \omega_1 - \rho\omega_2 J(\phi_1, \phi_2; \beta) - \rho\alpha\omega_1\omega_2 K(\phi_1, \phi_2; \beta), \tag{S12}$$

$$\dot{\phi}_2 = \omega_2 - \rho\omega_1 J(\phi_2, \phi_1; \beta) - \rho\alpha\omega_1\omega_2 K(\phi_2, \phi_1; \beta), \tag{S13}$$

where now $\rho = 3a\zeta_w/8\ell$, $\alpha = \bar{\omega}\zeta_0\zeta_w/\lambda$, and

$$J(\phi_i, \phi_j : \beta) = -[(2A(\beta) + B(\beta))\cos(\phi_i - \phi_j) + B(\beta)\cos(\phi_i + \phi_j)] \tag{S14}$$

$$K(\phi_i, \phi_j : \beta) = (2A(\beta) + B(\beta))\sin(\phi_i - \phi_j) + B(\beta)\sin(\phi_i + \phi_j). \tag{S15}$$

For example, this means that the phase difference $\chi = \phi_2 - \phi_1$ and phase sum $\Phi = \phi_1 + \phi_2$ evolve according to

$$\dot{\chi} = (\omega_2 - \omega_1)[1 + \rho J(\phi_1, \phi_2; \beta)] - 2\rho\alpha\omega_1\omega_2(2A(\beta) + B(\beta))\sin(\chi), \tag{S16}$$

$$\dot{\Phi} = (\omega_1 + \omega_2)[1 + \rho(2A(\beta) + B(\beta))\cos\chi + \rho B(\beta)\cos\Phi] - 2\rho\alpha\omega_1\omega_2 B(\beta)\sin\Phi. \tag{S17}$$

To the first order in the small quantities $\Delta\omega = \omega_2 - \omega_1$ and ρ, and averaging over a "natural" timescale of the fast variable Φ we get

$$\dot{\chi} = \Delta\omega - 2\alpha\omega_1\omega_2\,\rho(2A(\beta) + B(\beta))\sin\chi. \tag{S18}$$

$$\langle\dot{\Phi}\rangle = (\omega_1 + \omega_2)\left[1 + \rho(2A(\beta) + B(\beta))\cos\chi\right]. \tag{S19}$$

These functions can be rewritten as

$$\dot{\chi} = \Delta\omega + \tilde{D}(\chi) \tag{S20}$$

$$\langle\dot{\Phi}\rangle = (\omega_1 + \omega_2) + \tilde{S}(\chi) \tag{S21}$$

where $\tilde{D}(\chi) = D_0\sin\chi$ and $\tilde{S}(\chi) = S_0\cos\chi$. As in [3], time has been rescaled according to the mean angular speed $\bar{\omega}$, and both ω_i are measured in units of $\bar{\omega}$. Rewriting Eq. (S18) in dimensional units yields

$$\dot{\chi} = \Delta\omega - \frac{3a}{4\ell}\frac{\zeta_0\zeta_w^2}{\lambda}\omega_1\omega_2\big[2A(\beta) + B(\beta)\big]\sin(\chi). \tag{S22}$$

This is of the form $\dot{\chi} = \Delta\omega - C\sin(\chi)$, with $C > 0$. If $|\Delta\omega| < C$ then a stable fixed point $\dot{\chi} = 0$ exists. Conversely, for $|\Delta\omega| > C$, a cycle-averaged phase drift will occur. We use the following relation

$$\int_0^{2\pi}\big[a - b\sin\chi\big]^{-1}d\chi = \frac{2\pi}{\sqrt{a^2 - b^2}}, \quad \text{for } |a| > |b| \tag{S23}$$

to find the time-averaged phase drift

$$\dot{\chi}_{\rm av} = \sqrt{(\Delta\omega)^2 - \left(\frac{3a}{4l}\frac{\zeta_0\zeta_w^2}{\lambda}\omega_1\omega_2\big[2A(\beta)+B(\beta)\big]\right)^2}. \tag{S24}$$

For each rotor (indexed by $i \in \{0,1\}$), the intrinsic angular frequency is given by $\omega_i = F_i/(\zeta_0\zeta_w R_0)$ and so the above equations reads:

$$\dot{\chi}_{\rm av} = \sqrt{\left(\frac{F_1-F_0}{R_0\zeta_0\zeta_w}\right)^2 - \left(\frac{3a}{4\ell}\frac{F_0F_1}{\lambda\zeta_0R_0^2}\big[2A(\beta)+B(\beta)\big]\right)^2}. \tag{S25}$$

Since a detuning factor D is included so that the driving force is $F_i = F_{\rm dr}D^{i-1/2}$, the above equation can be written as

$$\dot{\chi}_{\rm av} = \frac{F_{\rm dr}}{R_0\zeta_0}\sqrt{\frac{(D-1)^2}{D\,\zeta_w^2} - \left(\frac{3a}{4\ell}\frac{F_{\rm dr}}{\lambda R_0}\big[2A(\beta)+B(\beta)\big]\right)^2}. \tag{S26}$$

The threshold value of D beyond which the rotors' phase difference will drift can be calculated explicitly.

Varying chain length

In order to assess the generality of the results presented in the main text, we used numerical simulations to explore the effect of changing the number of rotors, N, present in the linear array (see. Fig. 1a). Figure S2 shows the average phase drift (measured in beats per beat) with respect to the first rotor, along chains of different length, $N \in \{2, 15\}$. Each chain has a fixed detuning of 5% between the end rotors. For each height $h = 10\,\mu$m and $h = 100\,\mu$m, simulations were conducted with full hydrodynamic coupling and nearest neighbor coupling only.

The results for $N = 6$ are representative of the dynamics across a range of chain lengths. For chains in which rotors are coupled through nearest neighbor interactions, the rotors tend to phase-lock in clusters of 2-5 rotors. As discussed in the main text, the nearest neighbor results are fairly insensitive to changes in h, shown here by the similarly between the results of Fig. S2b and d. In stark contrast, the chains in which rotors are fully coupled to one another through hydrodynamic interactions exhibit qualitatively different behavior at different heights.

Truncation of hydrodynamic interactions

Figure S3 shows the results of deterministic numerical simulations, with hydrodynamic interactions truncated to be nearest neighbor in nature (see also Fig. 4d). The dynamics are almost completely insensitive to changes in h, across several orders of magnitude.

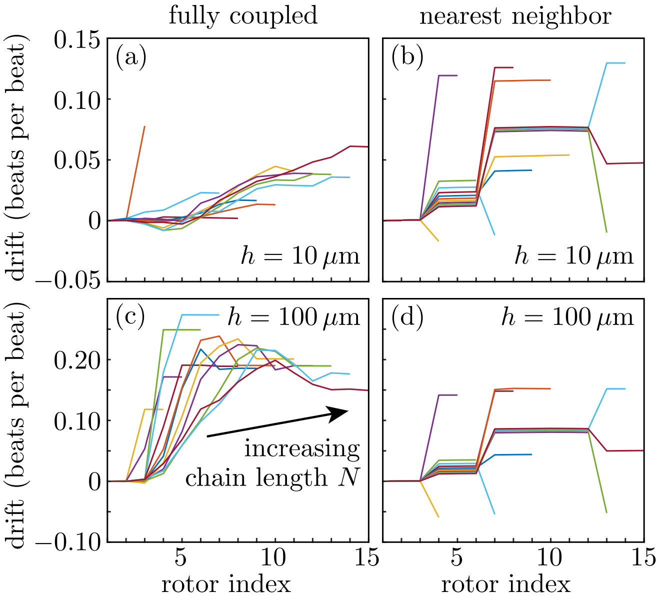

FIG. S2. (color online). Average phase drift with respect to the first rotor, for chains of different lengths $N \in \{2, 15\}$, at two different heights $h \in \{10\,\mu\text{m}, 100\,\mu\text{m}\}$, and subject to either full hydrodynamic coupling or nearest neighbor interactions only. The end-to-end detuning is fixed at 5% in each case, the radial spring stiffness is $\lambda = 4.5\,\text{pN}/\mu\text{m}$, and all other parameters are as in Fig. 4.

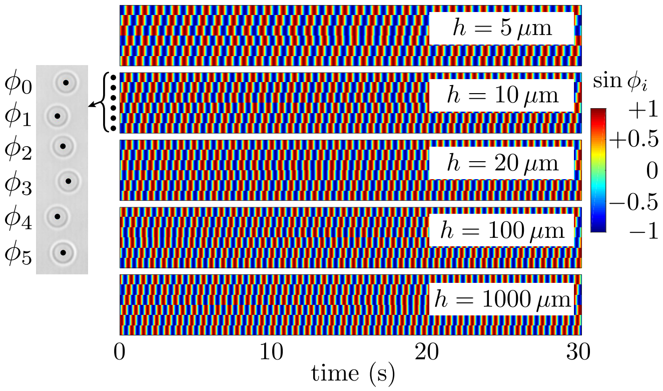

FIG. S3. (color online). Kymographs showing the phase $\sin\phi_i$ along the linear chain of model rotors, coupled hydrodynamically through the Blake tensor, but with interactions artificially restricted to be nearest neigbor. The radial spring stiffness is $\lambda = 4.5\,\text{pN}/\mu\text{m}$ and all other parameters are as in Fig. 4.

* M.Polin@warwick.ac.uk

[1] Vilfan, A. and Jülicher, F. (2006) *Physical Review Letters* **96(5)**, 58102.
[2] Blake, J. R. (1971) *Mathematical Proceedings of the Cambridge Philosophical Society* **70(2)**, 303–310.

[3] Niedermayer, T., Eckhardt, B., and Lenz, P. (2008) *Chaos* **18(3)**, 37128.
